# A review for the definition of the concept and symptoms of Hwa-Byung

**DOI:** 10.1192/j.eurpsy.2021.1803

**Published:** 2021-08-13

**Authors:** E. Kang, S. Lee, S. Choi

**Affiliations:** Clinical Psychology, Duksung Women’s University, seoul, Korea, Republic of

**Keywords:** Hwa-Byung, Literature research, Korean oriental psychiatry, Characteristic symptom

## Abstract

**Introduction:**

Hwa-Byung is a unique syndrome based on social-cultural background of South Korea. However, the definition of Hwa-Byung has not been established. For example, Hwa-Byung Diagnostic Interview Schedule(Kim, Kwon, Lee & Park, 2004) and Hwa-Byung Scale(Kwon et al., 2008) that are generally used in clinical practices defined Hwa-Byung differently. According to this, there is a slight difference in the symptoms that are measured.

**Objectives:**

The purpose of our study is to establish the concept and symptoms of Hwa-Byung.

**Methods:**

First, we review DSM-4, previous literatures and concept of Hwa-Byung in assessment tools. Through this, core features and characteristic symptoms are consisted. Second, a concept of Hwa-Byung that this study constructed is reviewed by clinical psychologists and Korean oriental psychiatrists. Finally, concepts and symptoms are defined.

**Results:**

Comprehensive definition of Hwa-Byung is established. Hwa-Byung was identified as a syndrome with symptoms that exploded in the form of anger because emotions such as anger could not be resolved. psychological symptoms include resentment, the baggage of mind, or a representative symptom of han. And physical symptoms include feeling heavy, heat, rush, lumps in the neck or chest. Finally, these physical and psychological symptoms are associated with distinct stressful events.

**Conclusions:**

Our study defined the concept and categorized for physical and emotional symptoms of Hwa-Byung. This result suggests that it can contribute to the development and revision of the Hwa-Byung assessment tools.

**Disclosure:**

No significant relationships.

